# An In Vitro Platform for the Rapid and Scalable Biocatalysis of Complex Polyketides

**DOI:** 10.21203/rs.3.rs-10935154/v1

**Published:** 2026-10-08

**Authors:** Jie Zhang, Adrian T. Keatinge-Clay

**Affiliations:** 1 Department of Molecular Biosciences, The University of Texas at Austin, Austin, TX

## Abstract

Modular polyketide synthases (PKSs) are enzymatic assembly lines that naturally produce complex, bioactive compounds challenging to obtain through traditional synthesis or fermentation. Early attempts to harness these molecular factories outside the cell indicated they make poor biocatalysts. However, we observe that a refactored pikromycin synthase optimized for in vitro performance operates as rapidly as iterative fatty acid synthases, two orders of magnitude faster than measured for the equivalently-sized erythromycin synthase. The activities of four engineered variants of the pikromycin synthase were also enhanced through relocating histidine tags, swapping enzymatic/docking domains, and tuning polypeptide stoichiometry. To enable the economic conversion of feedstock chemicals malonate and methylmalonate by these and other synthases into scalable quantities of complex polyketides, we developed the Extender unit to PolyKetide (EPK) platform, which incorporates extender unit and NADPH regeneration systems. In a 10-mL, 2-hour, room-temperature reaction, 1 μM of a refactored pikromycin synthase converts malonate and methylmalonte into 2.8 mM (1 mg/mL) narbonolide with a total turnover number (TTON) of 2800. All five stereocomplex products were readily obtained in pure form at the milligram scale and structurally characterized, demonstrating the advantages of accessing complex polyketide through in vitro biocatalysis as well as the potential to access combinatorial polyketide libraries for medicinal chemistry.

## INTRODUCTION

Modular polyketide synthases (PKSs) play the major role in the biosynthesis of many structurally complex, bioactive polyketides, such as the antibacterial erythromycin, the insecticide spinosyn, and the immunosuppressant rapamycin^1, 2^. Because the order of the catalytic domains in these enzymatic assembly lines largely determines the structures of their products, they can seemingly be re-engineered to access designer polyketides and combinatorial libraries for drug development. While synthetic chemists yearn to collaborate with these molecular factories to rapidly forge carbon–carbon bonds, install diverse functional groups, and set stereocenters under mild conditions, harnessing them remains a major challenge.

Most efforts to employ modular PKSs in the biocatalytic production of desired polyketides have been in vivo within *Streptomyces* or *E. coli* hosts (Fig. 1a)^3–6^. In vivo biocatalysis has been historically preferred to in vitro biocatalysis, as cells generate otherwise costly building blocks and coenzymes. However, the activities of assembly lines within cells are affected by many difficult-to-control factors, such as polypeptide stoichiometry, protein stability, building block supply, coenzyme supply, and competing metabolic pathways^7^. Thus, when low titers of desired polyketides are obtained, it can be difficult to determine whether it is the fault of the assembly line, the system housing it, or a combination thereof. While complex polyketides can be accessed through chemical synthesis, routes are usually compound-specific, resource-intensive, and low-yielding^8^.

In vitro biocatalysis provides a powerful means of studying, optimizing, and harnessing modular PKSs, as parameters such as polypeptide stoichiometries, substrate concentrations, and reaction conditions can be investigated and optimized for polyketide production. The first measurement of an in vitro reconstituted assembly line was of an engineered version of the 7-module erythromycin PKS (updated modules used throughout)^9, 10^ and indicated that PKSs are quite slow, with a *k_app_* (system turnover rate) of 1.1 min^−1^ (Fig. 1b)^11^. Our lab subsequently measured the 3-module venemycin PKS to operate with a *k_app_* of 37 min^−1^^12^. Whether this difference is from the smaller size of the venemycin PKS, how the erythromycin PKS was refactored, or reaction conditions was unclear. Encouragingly, milligram quantities of venemycin and similar polyketides were obtained through in vitro biocatalysis with the help of an extender unit regeneration system^12, 13^. As our lab recently accessed the heptaketide narbonolide through in vivo biocatalysis using a refactored version of the pikromycin PKS in *E. coli*, we sought to perform in vitro biocatalysis with this 7-module synthase and engineered variants to obtain semipreparative quantities of narbonolide and related complex polyketides^4, 14^.

Here, we demonstrate the rapid, scalable, and economic in vitro biocatalysis of 5 complex polyketides (**1–5**). The activities of their assembly lines were first optimized through tuning reaction conditions, swapping docking/enzymatic domains, repositioning histidine tags, supplying the editing thioesterase PikAV, and adjusting polypeptide stoichiometries. This resulted in a *k_app_* of 112 ± 11 min^−1^ (4 μM synthase) for the pikromycin PKS – 2 orders of magnitude faster than observed for the equivalent erythromycin PKS. We then developed the Extender unit to PolyKetide (EPK) platform, which includes extender unit and NADPH regeneration systems, to economically and rapidly obtain milligram quantities of each product and demonstrate the accessibility of diverse complex polyketides.

## RESULTS

### In vitro reconstitution of a refactored pikromycin synthase and engineered variants

We first aimed to in vitro reconstitute a refactored version of the pikromycin PKS that had been engineered for the in vivo biocatalytic production of narbonolide (**1**) (Figs. 1b–c and Extended Data Figs. 1 and 2a)^4^. While the natural synthase is composed of 4 polypeptides (PikAI–PikAIV), this refactored synthase is split by docking domains from the erythromycin and spinosyn PKSs into 6 polypeptides short enough to be expressed well in *E. coli* K207-3 (**P1P2**^N^, ^C^**P2P3**^N^, ^C^**P3P4**^N^, ^C^**P4P5**^N^, ^C^**P5P6**^N^, and ^C^**P6P7**; **P1–P7** are the pikromycin PKS modules, while ^N^ and ^C^ respectively indicate their AT+(KR/DH/ER)+ACP and KS regions; domains defined in Fig. 1)^15^. Thus, C-terminally His_6_-tagged versions these polypeptides were purified from this metabolically-engineered strain of *E. coli* that expresses a phosphopantetheinyl transferase to activate acyl carrier protein (ACP) domains (Fig. S1 and Table S1)^16, 17^.

Initial reactions were performed at 2 μM synthase with 1.2 mM (2*RS*)-methylmalonyl-CoA, 0.2 mM malonyl-CoA, and 1.2 mM NADPH (the theoretical ratio for narbonolide synthesis is 6:1:5) in 400 mM potassium phosphate (pH 7.5), using 5 mM tris(2-carboxyethyl)phosphine (TCEP) as a reductant (Figs. 1c, S2, and Extended Data Fig. 2b). An excess of NADPH was provided to both drive polyketide synthesis and compensate for its background oxidation. Time-course analysis showed that **1** increased during the first 8 min and decreased thereafter, suggesting its degradation. When **1** was incubated with individual reaction components, LC/MS analysis revealed its disappearance in the presence of TCEP as well as the concomitant appearance of peaks consistent with narbonolide–TCEP adducts (Extended Data Figs. 2c–f and Fig. S3). The related enone-containing macrolactone 10-deoxymethynolide (10-dml, **2**) is also degraded by TCEP, whereas hexaketide **3**, which does not contain an enone, is not sensitive to TCEP. Narbonolide (**1**) is also slowly degraded in 400 mM potassium phosphate, pH 7.5.

The *k_app_* of the refactored pikromycin PKS (**NAR_1_**) is 21 ± 3 min^−1^ (2 μM synthase) in the absence of TCEP (Extended Data Fig. 2b). After 4 min, nearly all supplied substrates are consumed, and 178 ± 8 μM **1** is generated (89% conversion). Complete conversion is precluded by the hydrolysis of malonyl-CoA by methylmalonyl-specific ATs, the competing synthesis of byproducts such as pentaketide pyrone **6** and triketide lactone **7**, and by the degradation of **1** in phosphate (Fig. S4, *vide infra*). Lower levels of byproducts are generated compared to in vivo biocatalysis^4, 7^. **NAR_1_** activity was examined across a range of conditions, including different buffer compositions, pH values, glycerol concentrations, and temperatures (Extended Data Fig. 3). The synthase is most active at pH 7.5 and essentially inactive at pH 6.0. The optimal temperature of 31 °C corresponds to the preferred growth temperature of its native host, *Streptomyces venezuelae* ATCC 15439. Since the apparent *K_d_* for the 2 polypeptides of the venemycin PKS had been measured to be 5 μM, how synthase concentration affects *k_app_* of the 6-polypeptide **NAR_1_** was examined^12^. It was observed to increase with synthase concentration (6 ± 1, 11 ± 1, 21 ± 2, and 35 ± 4 min^−1^ at 0.5, 1, 2, and 4 μM synthase, respectively), consistent with the formation of higher-order architecture (Fig. 1c)^18, 19^.

Since narbonolide (**1**) could be obtained from **NAR_1_**, we suspected other complex polyketides may be accessible from related, engineered synthases (Fig. 2). Indeed, anticipated polyketides are produced by each of the 4 synthases examined: the hexaketide macrolactone 10-dml (**2**) is produced by **DML_1_**, engineered by inactivating the **P6**KS active site cysteine of **NAR_1_** [*k_app_*: 1.3 ± 0.2 min^−1^ (2 μM synthase)]; the hexaketide δ-lactone **3** is produced by **ΔP3_1_**, engineered by deleting **P3** of **NAR_1_** [*k_app_* of 0.40 ± 0.04 min_−1_ (2 μM synthase)]; the hexaketide macrolactone **4** is produced by **ΔP6_1_**, engineered by deleting **P6** of **NAR_1_** [*k_app_* of 1.3 ± 0.3 min_−1_ (2 μM synthase)]; and the heptaketide macrolactone anhydronarbonolide (**5**)^20^ is produced by **ANH_1_**, engineered by swapping **P6** of **NAR_1_** for **R5** of the rapamycin PKS [*k_app_* of 0.40 ± 0.04 min_−1_ (2 μM synthase)]. As these synthases operate at only 2–6% the rate of **NAR_1_**, competing reactions are more apparent – significant quantities of byproducts are generated by ACP-independent condensations between methylmalonyl-CoA and KS-bound intermediates (Extended Data Fig. 4 and Figs. S4–5), and a large fraction of malonyl-CoA is nonproductively hydrolyzed by methylmalonyl-specific ATs (Extended Data Figs. 4–5). The omission of polypeptides from **NAR_1_** revealed byproducts **6** and **7** are respectively generated from the offloading of the **P2**KS-bound diketide and **P4**KS-bound tetraketide by methylmalonyl-CoA, similar to in vitro studies of polypeptides from the erythromycin synthase in which KS-bound intermediates were offloaded by fluoromalonyl-CoA^21^. While hydrolysis of malonyl-CoA by methylmalonyl-specific ATs had been previously observed, the extent of this side-reaction was surprising^22^. Malonyl-CoA added to **ΔP3_1_**, a synthase comprised of only methylmalonyl-specifice ATs, is consumed 5 times more rapidly than methylmalonyl-CoA.

### Modular PKSs can operate as fast as FASs

We sought to engineer versions of each assembly line that outpace competing reactions, beginning with the refactored pikromycin synthase (Extended Data Fig. 6). Since in vivo biocatalysis of narbonolide is improved by employing the native pikromycin docking domain motifs^4^, we reintroduced **P3**DD, **P5**DD, and **P6**DD. The *k_app_* of the resulting assembly line, **NAR_2_**, is 29 ± 4 min_−1_ (1 μM synthase), 2.4-fold greater than **NAR_1_**. Removing the docking domain motifs that had been introduced into **P4**, which does not natively contain them, had no effect on assembly line activity [*k_app_* (**NAR_3_**): 29 ± 2 min_−1_ (1 μM synthase)]. Since both termini participate in docking interactions, the locations of histidine tags were investigated with **NAR_4_–NAR_7_**. These designs revealed that tag relocation from the C-terminus to the loop between the KS and AT domains slightly improves activity [*k_app_* (**NAR_7_**): 37 ± 3 min_−1_ (1 μM synthase)]. Splitting the first polypeptide to facilitate protein purification did not significantly affect synthase activity [*k_app_* (**NAR_8_**): 36 ± 4 min_−1_ (1 μM synthase)] (Figs. 3a–b). As with **NAR_1_**, the rate of narbonolide production by **NAR_8_** increases with synthase concentration, plateauing at *k_app_* of 85 ± 11 min_−1_ with 4 μM synthase at 31 °C (Fig. 3c). The natural, 3-module venemycin PKS was re-examined and observed to plateau at a *k_app_* of 43 ± 2 min_−1_ with 2 μM synthase (Extended Data Fig. 7).

Premature decarboxylation of ACP-bound extender units results in acetyl and propionyl intermediates that stall assembly lines. As the addition of the editing thioesterase PikAV that removes these groups improved in vivo biocatalysis by the refactored pikromycin PKS, it was investigated^4, 23^. The *k_app_* of **NAR_1_** (2 μM synthase) increases 1.5-fold in the presence of 1.75–7 μM PikAV (Figs. 3d and S6). Supplying 3.5 μM PikAV to **NAR_8_** increases its activity 1.3-fold, enabling the highest *k_app_* observed in this study: 112 ± 11 min_−1_ (4 μM synthase). This is equivalent to several well-studied, iterative type I fatty acid synthases (FASs; *vide infra*)^24–27^. Under these conditions, 0.9 mM narbonolide is formed from 1 mM malonyl-CoA in 3 min (90% conversion).

Although relocating histidine tags to the loop between KS and AT yielded the most active synthases, C-terminally-tagged polypeptides are more readily purifiable. Thus, the purification-friendly, 7-polypeptide **NAR_9_** was engineered [*k_app_* (**NAR_9_**): 27 ± 5 min_−1_ (1 μM synthase)] (Fig. 3a). This design was selected for the scaled-up in vitro biocatalysis of narbonolide and as the basis for redesigning the **DML, ΔP3, ΔP6**, and **ANH** synthases (Extended Data Fig. 8).

### Optimizing domain compositions

**DML_1_, ΔP3_1_, ΔP6_1_**, and **ANH_1_** are 94–98% less active than **NAR_1_**, the synthase from which they are derived. This is likely a result of suboptimal domain-domain and polyketide-domain interactions. We sought to learn how to optimize domain-domain interactions using the in vitro platform by studying model synthases in which polyketide-domain interactions are removed from the equation. The 6^th^ modules of the pikromycin and erythromycin PKSs, **P6** and **E6**, perform identical chemistry on hexaketide intermediates possessing identical α–ζ substituents (Fig. 4a). Thus, assembly lines in which domains in **P6** and the surrounding region were swapped with equivalent domains from the erythromycin PKS were combinatorially constructed. KSs and the processing enzymes upstream of them are now known to closely evolutionarily co-migrate and are grouped together within the updated module^9, 10^; KSs physically associate with the acyl-ACPs upstream of them to enable the transacylation of correctly processed acyl chains^28, 29^. The synthase in which **P5**KS, **P6**AT, **P6**KR, and **P6**ACP (the traditional module, **x_1_y_1_**) are swapped contains 2 disrupted ACP/KS interfaces and is one of the least active. Although the synthase in which **P6**AT, **P6**KR, **P6**ACP, and **P6**KS (the updated module, **x_2_y_2_**) are swapped was quite active, the most active synthase was constructed by swapping only **P6**KR, **P6**ACP, and **P6**KS (**x_3_y_2_**). The synthase in which **P6**KR, **P6**ACP, **P6**KS, and **P7**AT (equivalent to the “XUA” set of domains that yielded the best results in recent engineering of the mediomycin PKS, **x_3_y_3_**) are swapped is much less active^5^. The domain-swapping strategy was also applied to optimize **ANH_1_** and yielded equivalent results – swapping **P6**(KR+ACP+KS) for **R5**(DH+KR+ACP+KS) (**x_2_y_2_**) boosts productivity 2.5-fold (Fig. 4b).

The Sherman lab observed 10-dml (**2**) production from incubating a pentaketide substrate with PikAIII and a version of PikAIV in which the **P6**KS reactive cysteine is mutated to alanine. The production rate increased when the **P7**ACP serine that is phosphopantetheinylated is also mutated to alanine. We observe this mutation to enhance activity 9.3-fold [*k_app_* (**DML_3_**): 6.5 ± 0.9 min_−1_] (Extended Data Figure 8). However, the deletion of **P7**ACP enhances activity 11-fold [*k_app_* (**DML_4_**): (7.6 ± 0.9 min_−1_)]. With **DML_4_** at 2 μM and PikAV at 3.5 μM, *k_app_* is 13.6 ± 1.4 min_−1_.

### Increased flux through stoichiometric tuning

A general practice in metabolic engineering is to increase the concentration of enzymes suspected to be at the bottleneck of a biochemical pathway. Thus, we examined the effect of increasing the concentrations of the assembly line polypeptides most affected through the engineering performed to generate the **DML, ΔP3, ΔP6**, and **ANH** synthases. The concentrations of the 3^rd^ and 4^th^ polypeptides of the module-deleted synthase **ΔP3_3_, ^C^P2P4^N^** and **^C^P4P5^N^**, were varied while maintaining the others at 1 μM (Fig. 5a and Extended Data Fig. 8). Activity is not enhanced by independently increasing the concentration of the upstream polypeptide, **^C^P2P4^N^**, but is enhanced by independently increasing the concentration of the downstream polypeptide, **^C^P4P5^N^**. Interestingly, when the concentration of the downstream polypeptide, **^C^P4P5^N^**, is increased, activity can be further enhanced by increasing the concentration of the upstream polypeptide, **^C^P2P4^N^**, but only until it equals the concentration of the downstream polypeptide, **^C^P4P5^N^**. With 4 μM ^C^**P2P4**^N^ and 8 μM ^C^**P4P5**^N^, the *k_app_* for **ΔP3_3_** is enhanced 4-fold to 12.5 ± 1.2 min_−1_. The same trend was observed when titrating the 6^th^ and 7^th^ polypeptides of **ANH_3_**: its *k_app_* is enhanced 12-fold with 8 μM ^C^**P5R5**^N^ and 16 μM ^C^**R5P7** to 16.9 ± 1.1 min_−1_ (Fig. 5b). The activities of **DML_4_** and **ΔP6_3_** were not enhanced by increasing the concentrations of their 2 downstream polypeptides.

### In vitro biocatalysis yields semi-preparative quantities of diverse complex polyketides

To enable scaled-up biocatalytic reactions with **NAR_9_, DML_4_, ΔP3_3_, ΔP6_3_**, and **ANH_3_**, the Extender unit to PolyKetide (EPK) platform was developed (Fig. 6). Its reaction mixture includes regeneration systems for complex polyketide synthesis – adenylate kinase, pyruvate kinase, MatB, and methylmalonyl-CoA epimerase for malonyl- and (2*S*)-methylmalonyl-CoA regeneration as well as glucose dehydrogenase for NADPH regeneration. Under platform conditions, malonyl-CoA can be produced at 31 μM min_−1_, and a 1:1 mixture of (2*R*)- and (2*S*)-methylmalonyl-CoA can be produced at 88 μM min_−1_ (ATP is supplied 4 min before the PKS polypeptides to generate the starting pool of extender units). These rates support narbonolide formation in a 100-μL reaction containing 1 μM **NAR_9_** and 3.5 μM PikAV at room temperature (23 °C).

Next, a 10-mL EPK reaction was performed to test the semi-preparative production of narbonolide (Fig. 6a). Its initial rate was equivalent to that of a reaction containing malonyl-CoA, (2*RS*)-methylmalonyl-CoA and NADPH, but no regeneration enzymes (43 versus 47 μM min_−1_), indicating that extender unit and coenzyme regeneration keep pace with the assembly line. The narbonolide (**1**) concentration reached 1 mg mL_−1_ (2.8 mM) after 2 h. Following ethyl acetate extraction and HPLC purification, 7.2 mg of pure narbonolide was obtained, enabling its characterization by NMR and x-ray diffraction (Fig. S10 and Extended Data Table 1). This is the first time the crystal structure of this iconic polyketide has been reported.

During 8-h reactions with slower synthases, GDH was observed to precipitate. However, after increasing its concentration to 10 μM and supplying 5% (v/v) glycerol, each of **DML_4_, ΔP3_3_, ΔP6_3_** and **ANH_3_** yielded their anticipated polyketides (**2–5**) in quantities sufficient for characterization by NMR (and x-ray crystallography for **4**; Figs. 6b and S11–21, Extended Data Table 1). The clean background of the in vitro reactions enabled the detection of unexpected products that could easily have been missed working up in vivo biocatalytic reactions. A compound observed while purifying **5** from the **ANH_3_** reaction was analyzed by LC/MS and NMR and determined to be the linear product **8** (Figures S27–31). Similarly, a compound observed while purifying macrolactone **4** from the **ΔP6_3_** reaction was determined to be its C2-epimer, **9** (Figures S32–36).

## DISCUSSION

Complex polyketides are currently accessed through chemical synthesis or fermentation rather than through in vitro biocatalysis. Early in vitro studies of modular PKSs led to the notion that harnessing them outside the cell is impractical: a reconstituted erythromycin synthase was measured to produce 6-deoxyerythronolide B at a sluggish 1.1 min^−1^^11^, and the highest observed TTON measured for PKS polypeptides was 300 for the conversion of a pentaketide substrate to narbonolide by PikAIII and PikAIV^30^. In addition these unimpressive biocatalytic parameters, potential deterrents to in vitro biocatalysis include the challenges of pathway engineering and polypeptide purification as well as the high costs of required substrates and coenzymes.

Here, we demonstrate that complex polyketides can be obtained through in vitro biocatalysis and that this route offers several advantages. Design and reaction optimization for the refactored pikromycin PKS led to **NAR_8_**, which can synthesize narbonolide with a *k_app_* of 112 ± 11 min_−1_, two orders of magnitude faster than measured for the equivalently-sized erythromycin PKS. Thus, each module in **NAR_8_** operates at least at 1.9 cycles per second, similar to rates measured for iterative, type I fatty acid synthases (FASs) from *Saccharomyces cerevisiae, Corynebacterium ammoniagenes*, and *Gallus gallus* at 18, 2.0, and 3.4 cycles per second, respectively^24–27^. While the reaction chambers of the structurally-characterized hexameric *S. cerevisiae* FAS and dimeric *Dictyostelium discoideum* are quite different, both contain relatively mobile ACP domains that transiently dock rigidly-oriented AT, KS, KR, DH, and ER domains^31, 32^. Thus, pikromycin synthase is likely comprised of highly organized reaction chambers, which overlap such that ACPs convey intermediates from one to the next. Here, we show PKSs are not only faster than expected but also more biocatalytically robust, possessing a half-life of ~1 day and capable of high turnover. For the 2-hour, 10-mL, room temperature EPK reaction in which 1 μM **NAR_9_** generates 2.8 mM narbonolide, the TTON is a solid 2800. EPK reactions are relatively inexpensive, as they only utilize catalytic quantities of ATP, CoA, and NADPH. Thus, the complex polyketide narbonolide can be accessed through in vitro biocatalysis more rapidly and economically than through chemical synthesis. Likewise, narbonolide can be accessed more rapidly through in vitro biocatalysis than in vivo biocatalysis; follow-up studies will determine whether it is also more economical^4^.

Synthases are quite sensitive to changes imparted through PKS engineering. The module deletions that yielded **ΔP3_1_** and **ΔP6_1_**, the module-swapping that yielded **ANH_1_**, and the point mutation that yielded **DML_1_** each result in suboptimal domain-domain and polyketide-domain interactions, leading to 94–98% decreases in activity relative to **NAR_1_**. Even the swapping of docking domains led to a 59% difference in activity between **NAR_2_**, which harbors the pikromycin docking domains, and **NAR_1_**, which harbors docking domains from the erythromycin and spinosyn PKSs. We describe several methods to boost the activities of engineered synthases, including determining the optimal set of domains for swapping and increasing the stoichiometries of the polypeptides most affected by the engineered change. While the best results from EPK reactions are obtained from synthases that operate with a *k_app_* of at least 10 min^−1^, even **ΔP6_3_**, which operates at 3.8 ± 0.5 min_−1_, yields 2.7 mg of pure hexaketide macrolactone **4** from a 20-mL EPK reaction.

The EPK platform reaches far beyond providing scalable access to a desired complex polyketide. As demonstrated, it can quantitatively measure assembly line activities independent of cellular metabolism to quickly provide feedback to design-build-test-learn PKS engineering cycles. The much smaller volumes and complexity enabled by the platform compared to in vivo biocatalysis greatly facilitate product purification and characterization by NMR and crystallography. It enables structural biology studies of PKS higher-order architecture through advanced techniques such as cryo-electron tomography and atomic force microscopy. The EPK platform could also aid in the synthesis of large, combinatorial libraries of complex polyketides through automated liquid handling of PKS polypeptides in multi-well plates to accelerate the medicinal chemistry needed to optimize complex polyketide drug leads. Thus, this biotechnological innovation has opened diverse collaborations with some of Nature’s most powerful synthetic factories.

### Reporting Summary

Further information on research design is available in the Nature Portfolio Reporting Summary linked to this Article.

## Extended Data

**Extended Data Figure 1.**
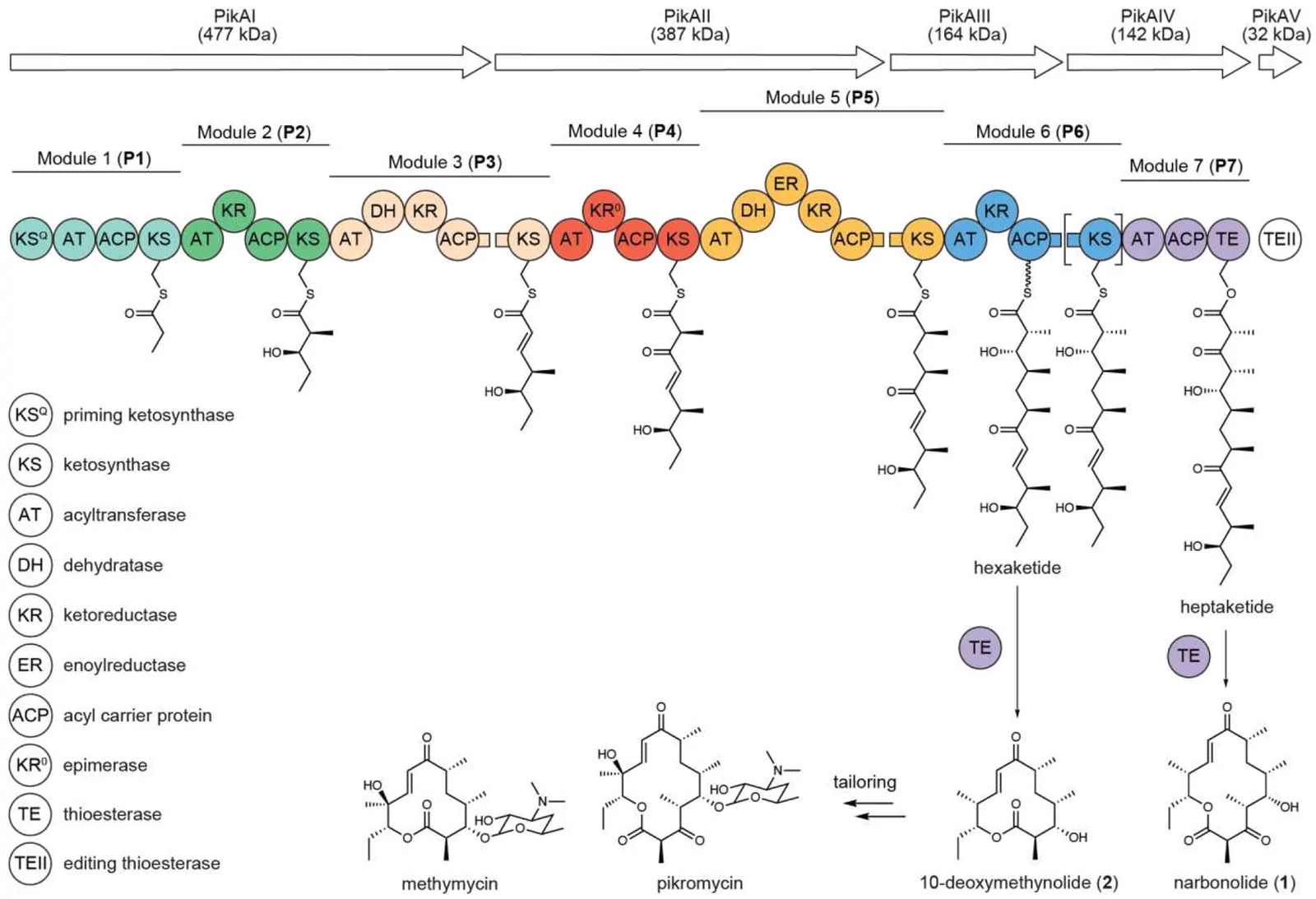
The pikromycin polyketide synthase (PKS) and its products. Four polypeptides house 7 modules (updated definition), **P1**–**P7**, that synthesize the macrolactones narbonolide (**1**) and 10-deoxymethynolide (10-dml, **2**), precursors of the macrolide antibiotics pikromycin and methymycin. Inactivation of the most downstream KS results in the production of 10-dml. Rectangles represent the docking domain motifs, ^C^DD and ^N^DD.

**Extended Data Figure 2.**
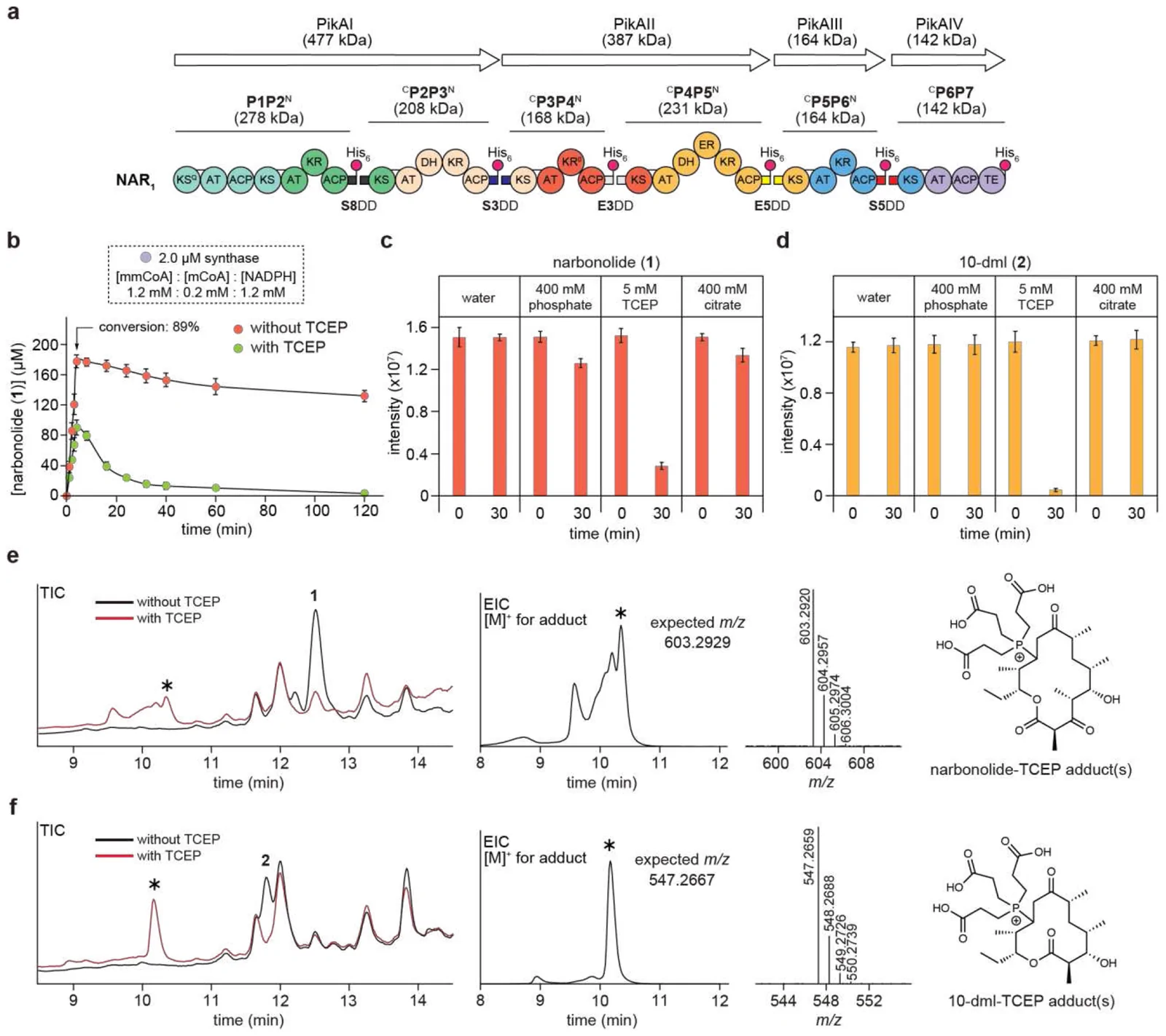
In vitro biocatalysis of narbonolide (**1**). a) His_6_-tags (pink circles) were engineered at the C-termini of the 6 polypeptides of a refactored pikromycin PKS that generates narbonolide in *E. coli* K207-3 to facilitate their purification. The polypeptides noncovalently assemble through docking domain (DD) motifs imported from modules of the spinosyn (**S**) and erythromycin (**E**) PKSs. b) Narbonolide produced by **NAR_1_** (2 μM synthase) disappears in reactions containing TCEP. c) Pure narbonolide is degraded rapidly by TCEP and slowly by phosphate and citrate. d) Pure 10-dml (**2**) is degraded rapidly by TCEP. New compounds are observed by LC/MS when (e) narbonolide (**1**) or (f) 10-dml (**2**) is incubated with TCEP for 30 minutes. TIC (total ion chromatogram), EIC (extracted ion chromatogram).

**Extended Data Figure 3.**
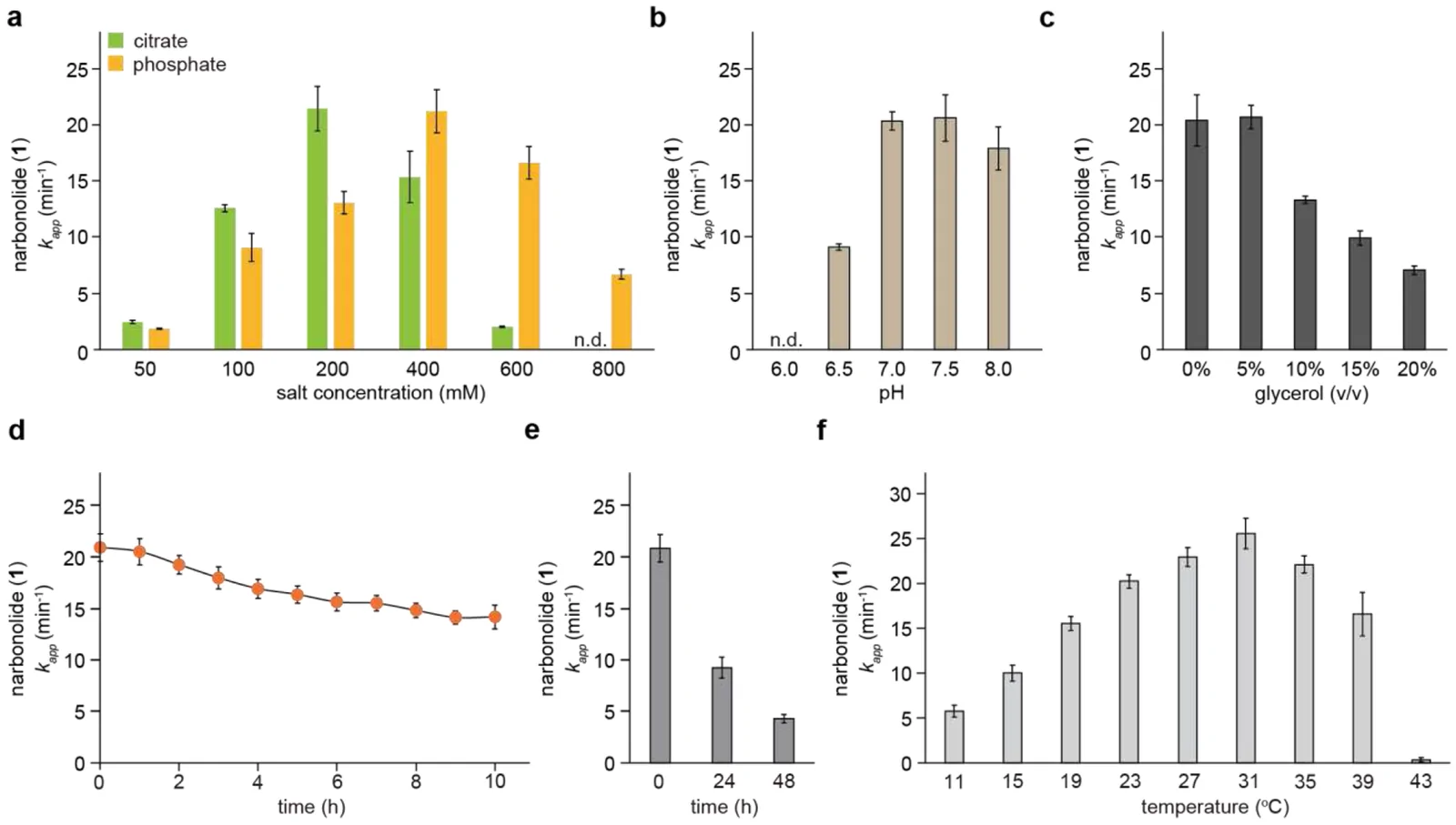
Determining the optimal conditions for narbonolide (**1**) synthesis. Production is optimal in a) 200 mM citrate or 400 mM phosphate, b) at pH 7.5, c) in 5% (v/v) glycerol, d) when the synthase is left at room temperature (23 °C) for hours or e) days, and f) at 31 °C.

**Extended Data Figure 4.**
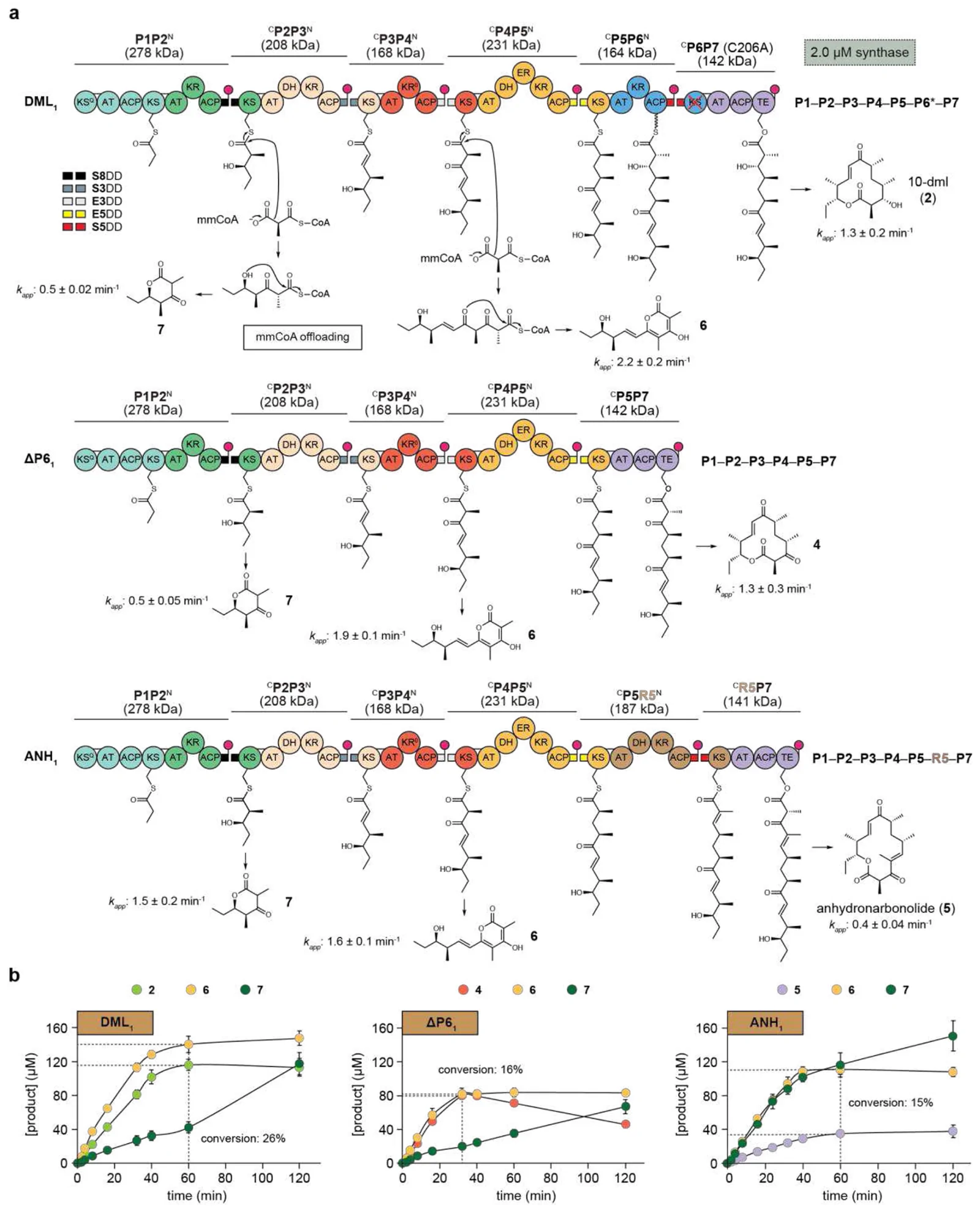
Product profiles of **DML_1_**, **ΔP6_1_**, and **ANH_1_**. The diketide intermediate bound to **P2**KS and the tetraketide intermediate bound to **P4**KS are susceptible to offloading by (2*S*)-methylmalonyl-CoA. The resulting generation of triketide **6** and pentaketide **7** competes with the synthesis of the desired polyketides, 10-dml (**2**), **4**, and anhydronarbonolide (**5**).

**Extended Data Figure 5.**
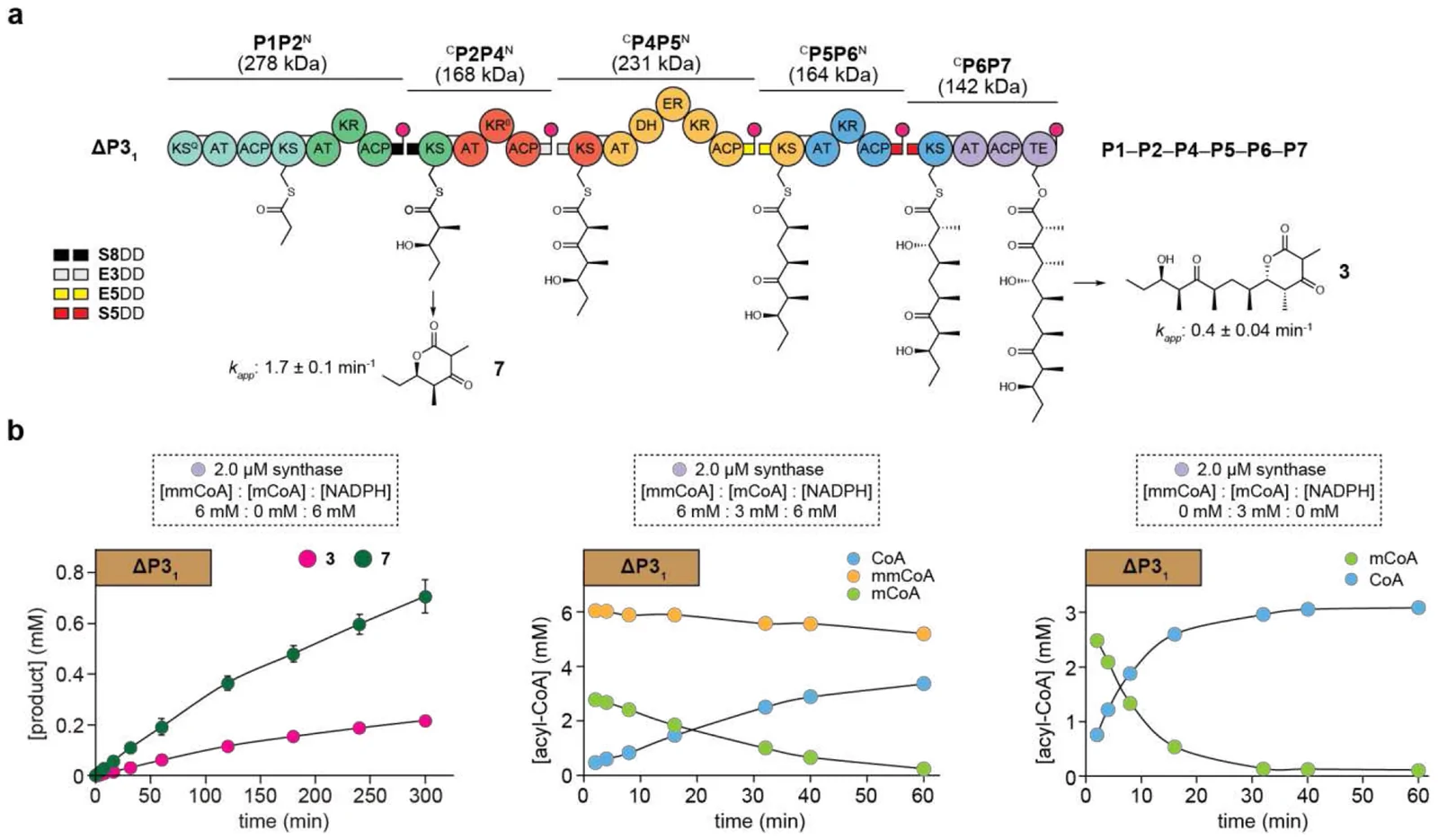
Reactions competing with the production of desired polyketide from a module-deleted PKS. a) **P1**-**P2**-**P4**-**P5**-**P6**-**P7** produces hexaketide **3** as well as byproduct **7** through methylmalonyl-CoA offloading. b) While this synthase does not contain any malonyl-specific ATs, malonyl-CoA supplied to the reaction is consumed 5 times faster than methylmalonyl-CoA. Malonyl-CoA hydrolysis by methylmalonyl-selective AT domains is even faster (*k_app_*: 100 min^−1^) in the absence of methylmalonyl-CoA.

**Extended Data Figure 6.**
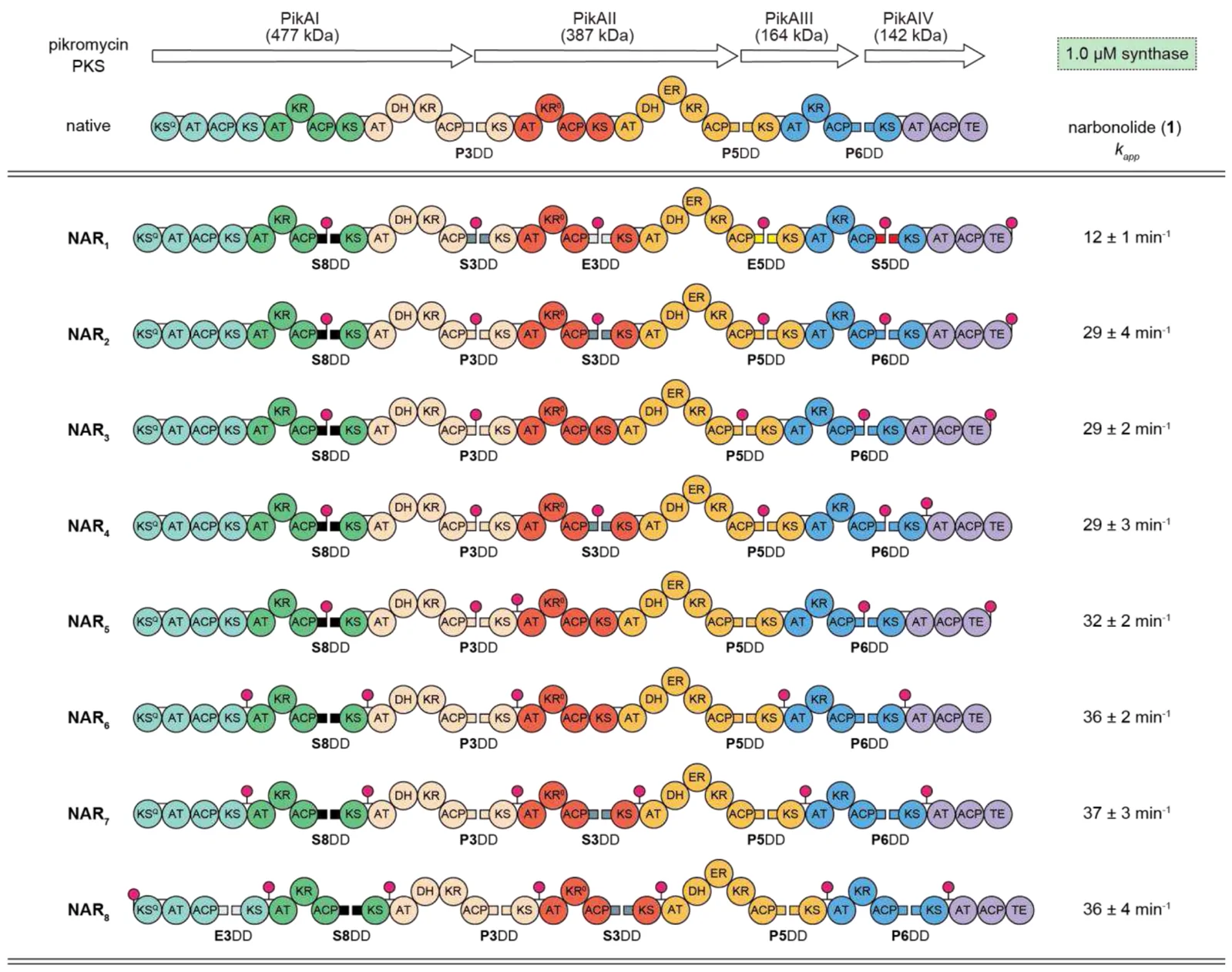
Synthase architecture affects narbonolide (**1**) production. **NAR_1_**– **NAR_8_** test how docking domains, His_6_-tags, and polypeptide boundaries influence PKS activity. Using the native pikromycin docking domains and relocating His_6_-tags from the C-terminus to the flexible loop between KS and AT improved PKS activity. Introducing docking domains to split polypeptides did not change PKS activity.

**Extended Data Figure 7.**
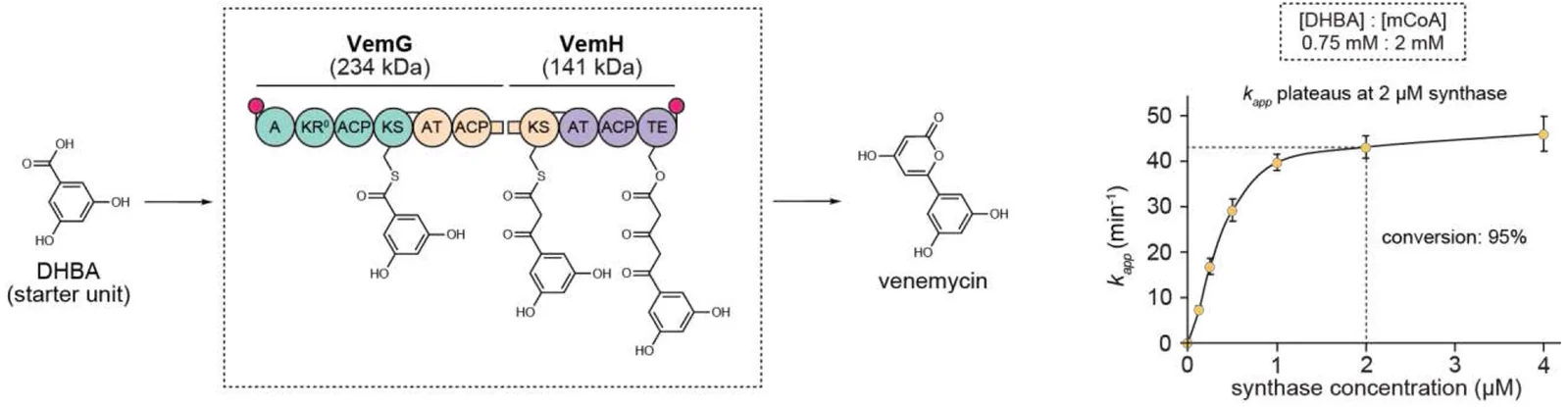
In vitro reconstitution of venemycin biosynthesis. Purified VemG and VemH were supplied with 3,5-dihydroxybenzoic acid (DHBA) and malonyl-CoA to generate venemycin. *k_app_* increased with synthase concentration and plateaued at ~2 μM synthase, reaching 43 ± 2 min^−1^ with ~95% conversion.

**Extended Data Figure 8.**
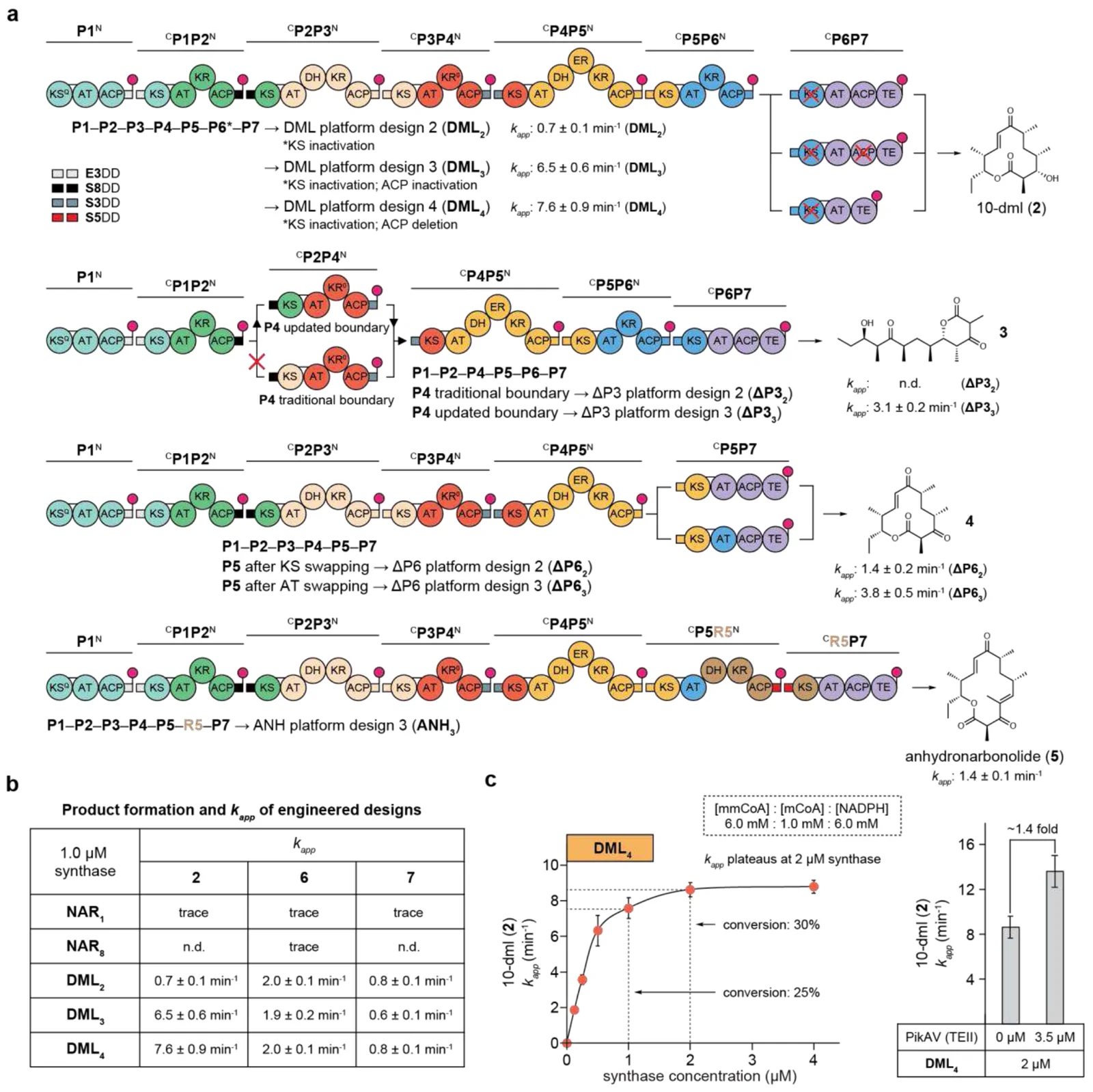
Optimizing assembly line designs. a) Rate measurements for versions of the engineered **DML**, **ΔP3**, **ΔP6**, and **ANH** synthases. Subsequent increases in *k_app_* were obtained through tuning polypeptide stoichiometries. b) Rates of product/byproduct formation for versions of **NAR** and **DML** at 1.0 μM synthase. While the rate of byproduct formation is similar between **DML_2_**–**DML_4_**, the rate of 10-dml (**2**) production is significantly greater for **DML_3_** and **DML_4_**, possibly because holo-**P7**ACP inhibited acyl-**P6**ACP docking to TE. c) The *k_app_* of **DML_4_** plateaus at 2 μM synthase, a slightly lower concentration than observed for **NAR_8_** (4 μM). The addition of thioesterase II (PikAV) increased the **DML_4_** *k_app_* 1.4-fold to 13.6 ± 1.4 min^−1^.

**Extended Data Table 1.** EPK reaction conditions, yields, and structural characterizations.

Compound	Platform	PKS concentration	EPK	PikAV	Isolated yield	Characterization
**1**	**NAR_9_**	1 μM all	EPK (10 mL, 2 h)	Yes	7.2 mg	NMR, X-ray
**2**	DML_4_	1 μM all	optimized EPK 5% (v/v) glycerol 10 μM GDH 20 mL 8 h	Yes	4.6 mg	NMR
**3**	**ΔP3_3_**	^C^**P2P4**^N^: 2 μM; ^C^**P4P5**^N^: 2 μM all others: 1 μM		No	3.6 mg	NMR
**4**	**ΔP6_3_**	1 μM all		No	2.7 mg	NMR, X-ray
**5**	**ANH_3_**	^C^**P5R5**^N^: 4 μM; ^C^**R5P7**: 16 μM all others: 1 μM		No	5.1 mg	NMR

## Supplementary Material

This is a list of supplementary files associated with this preprint. Click to download.

- Compound4.cif
- Compound1.cif
- Compound4CheckCIF.pdf
- SIsubmission.pdf
- Compound1CheckCIF.pdf

## Figures and Tables

**Figure 1.**
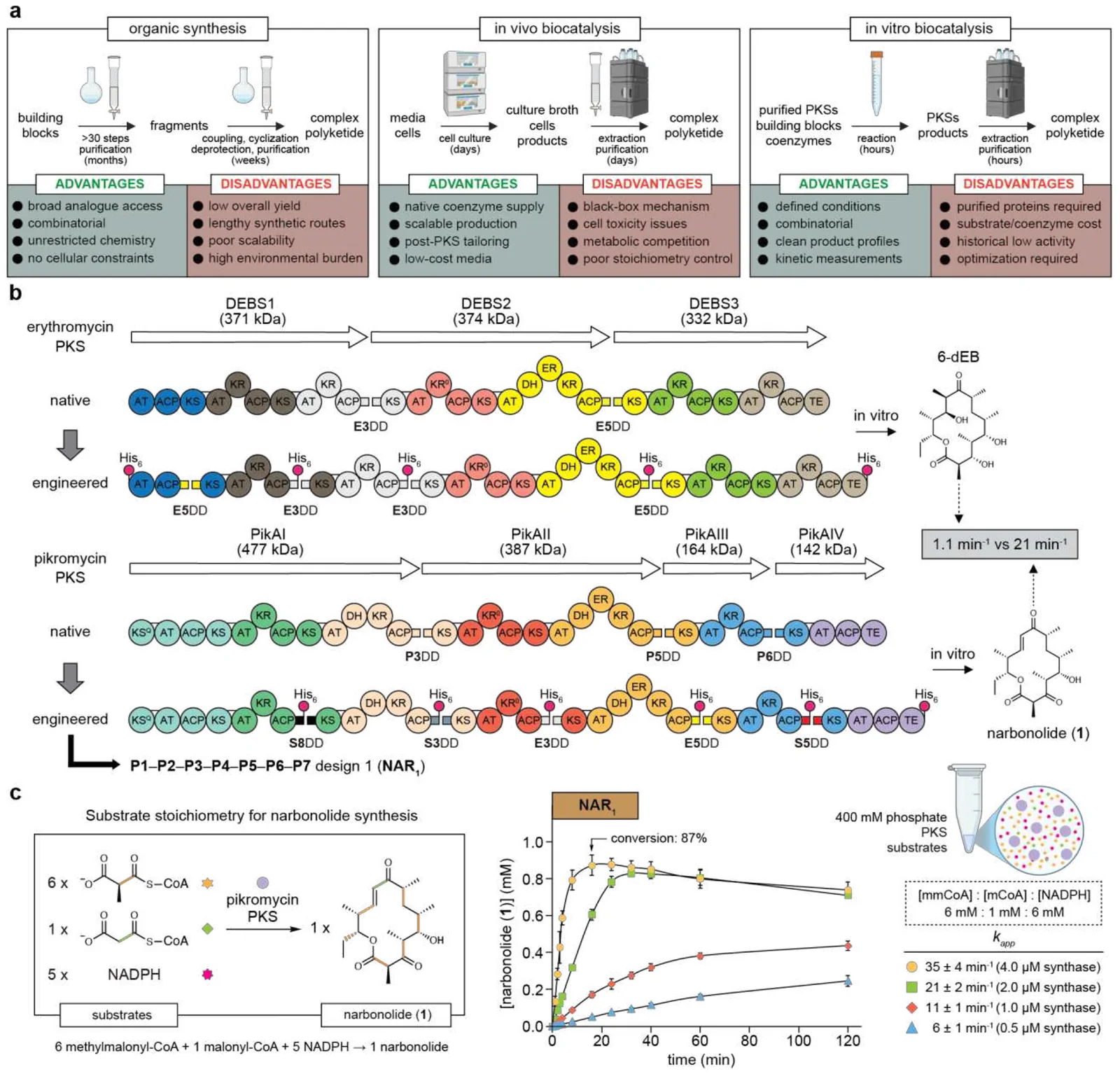
In vitro biocatalysis with modular PKSs. a) The advantages and disadvantages of 3 routes to accessing desired polyketides – chemical synthesis, in vivo biocatalysis, and in vitro biocatalysis – are compared. b) The first report of an in vitro reconstituted PKS was of an engineered version of the erythromycin PKS, which was measured to operate with a *k_app_* of 1.1 min^−1^ (2 μM synthase). The first version of the pikromycin PKS tested here (**NAR_1_**) operates with a *k_app_* of 21 min^−1^ (2 μM synthase). His_6_-tags and docking domains (**E**, erythromycin; **P**, pikromycin; **S**, spinosyn) are indicated. AT, acyltransferase; DH, dehydratase; ER, enoylreductase; ACP, acyl carrier protein; KS, ketosynthase; TE, thioesterase. c) (Methyl)malonyl-CoA substrates and NADPH coenzyme, supplied in slight excess, are rapidly converted to narbonolide by the pikromycin PKS (87% in 16 min). The dependence of *k_app_* on enzyme concentration is indicative of multimeric architecture.

**Figure 2.**
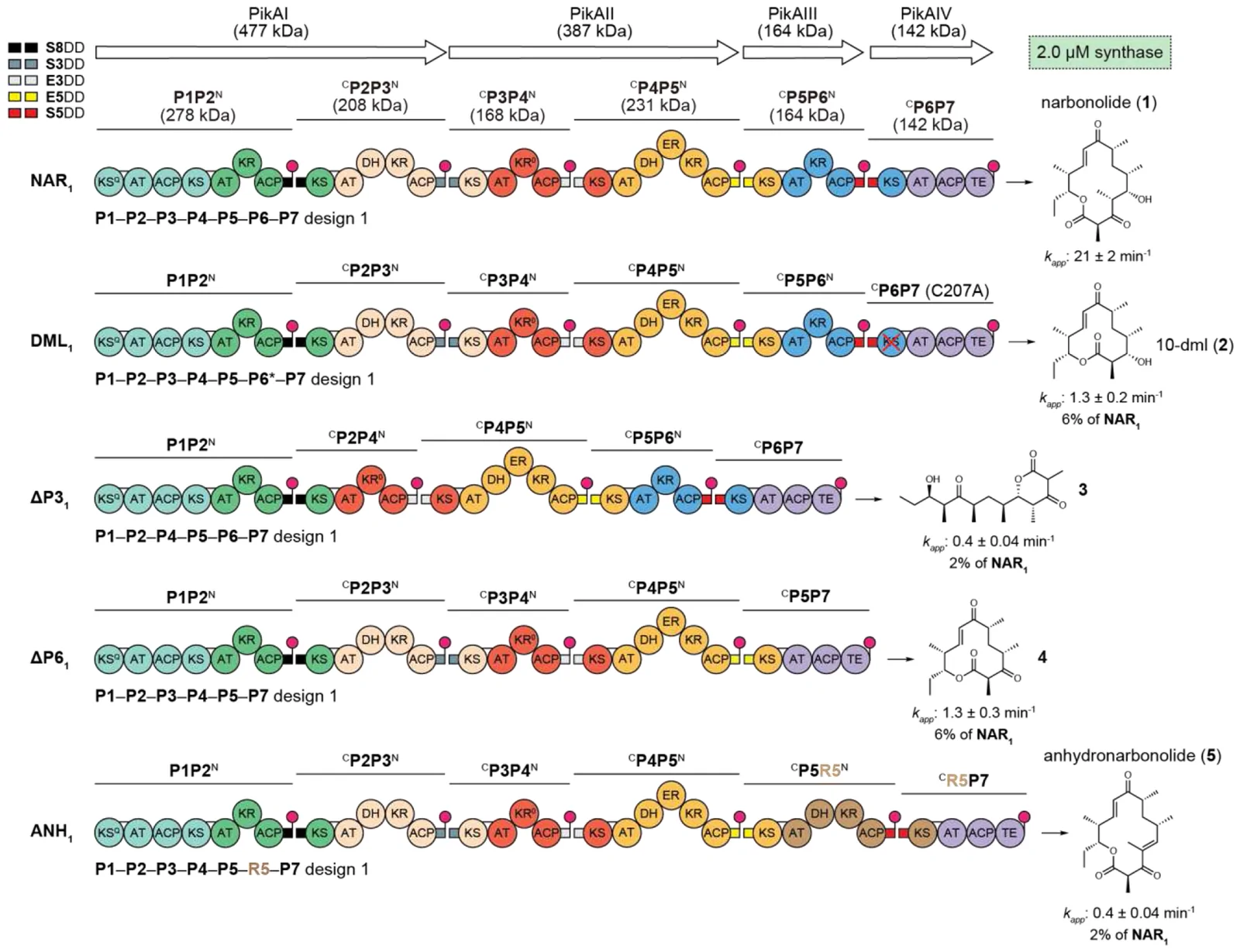
Initial designs for the pikromycin PKS and 4 engineered variants. These assembly lines produce heptaketides narbonolide (**1**) and anhydronarbonolide (**5**) as well as hexaketides 10-dml (**2**), **3**, and **4**. The *k_app_* values, measured for each initial design at 2 μM synthase, show the engineered variants are at least an order of magnitude slower than the refactored pikromycin PKS **NAR_1_**.

**Figure 3.**
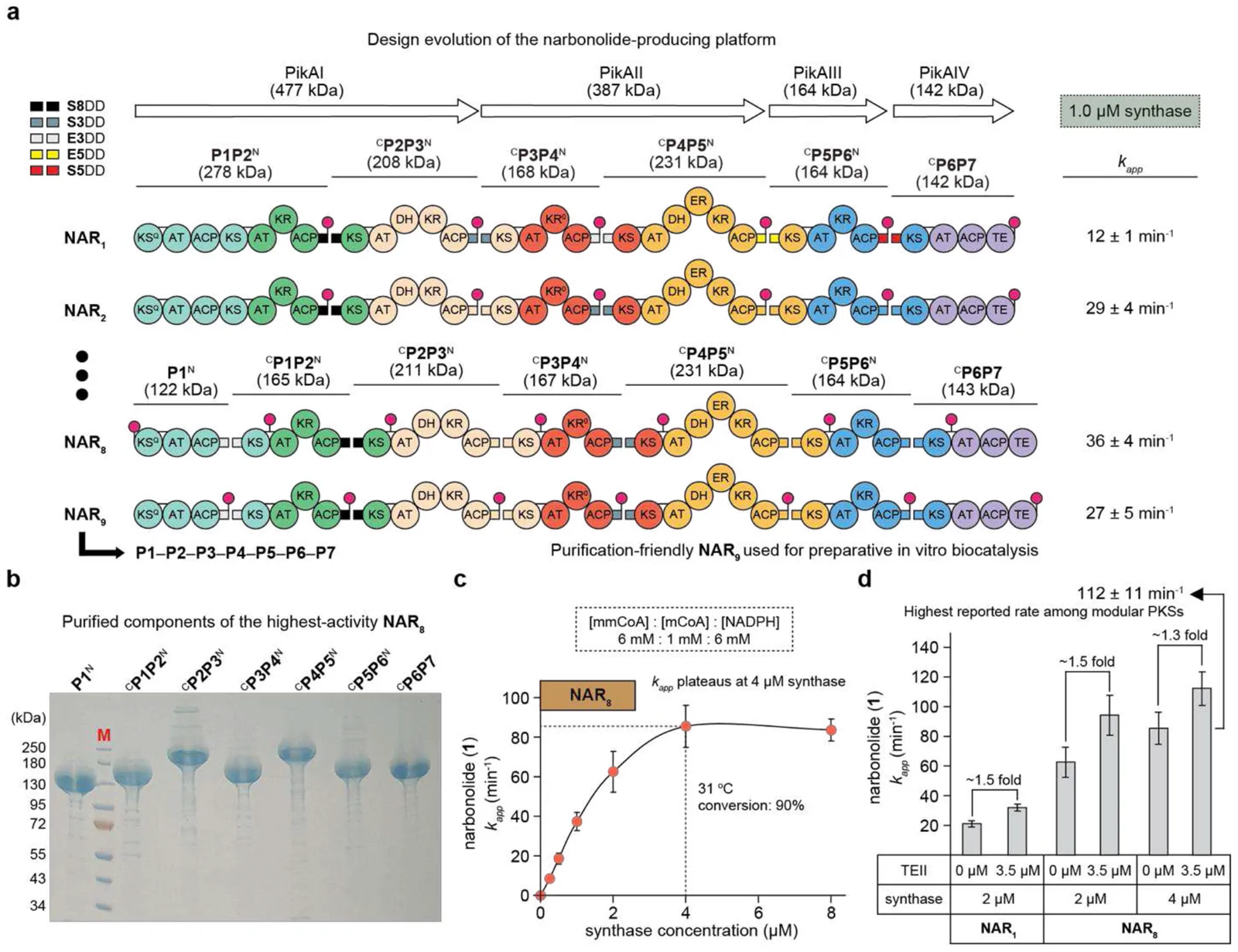
Optimizing pikromycin PKS for narbonolide production. a) Several versions of the pikromycin PKS, differing in polypeptide boundaries, docking domains, and histidine tag locations were engineered. Replacing heterologous docking domains with those native to the pikromycin PKS increased assembly line activity 140%. Relocating histidine tags to the loop between KS and AT increased activity 24%. While **NAR_8_** showed the highest turnover rate, the C-terminally tagged polypeptides of **NAR_9_** were more readily purified. Thus, **NAR_9_** was chosen for preparative polyketide production. b) SDS-PAGE analysis of the 7 purified polypeptides of **NAR_8_**. c) The *k_app_* of **NAR_8_** increases with [synthase] until 4 μM. The optimal temperature is 31 °C (see Extended Data Fig. 3f). d) In the presence of 3.5 μM PikAV, **NAR_8_** operates with a *k_app_* of 112 ± 11 min^−1^ (4 μM synthase), the highest rate measured for a modular PKS.

**Figure 4.**
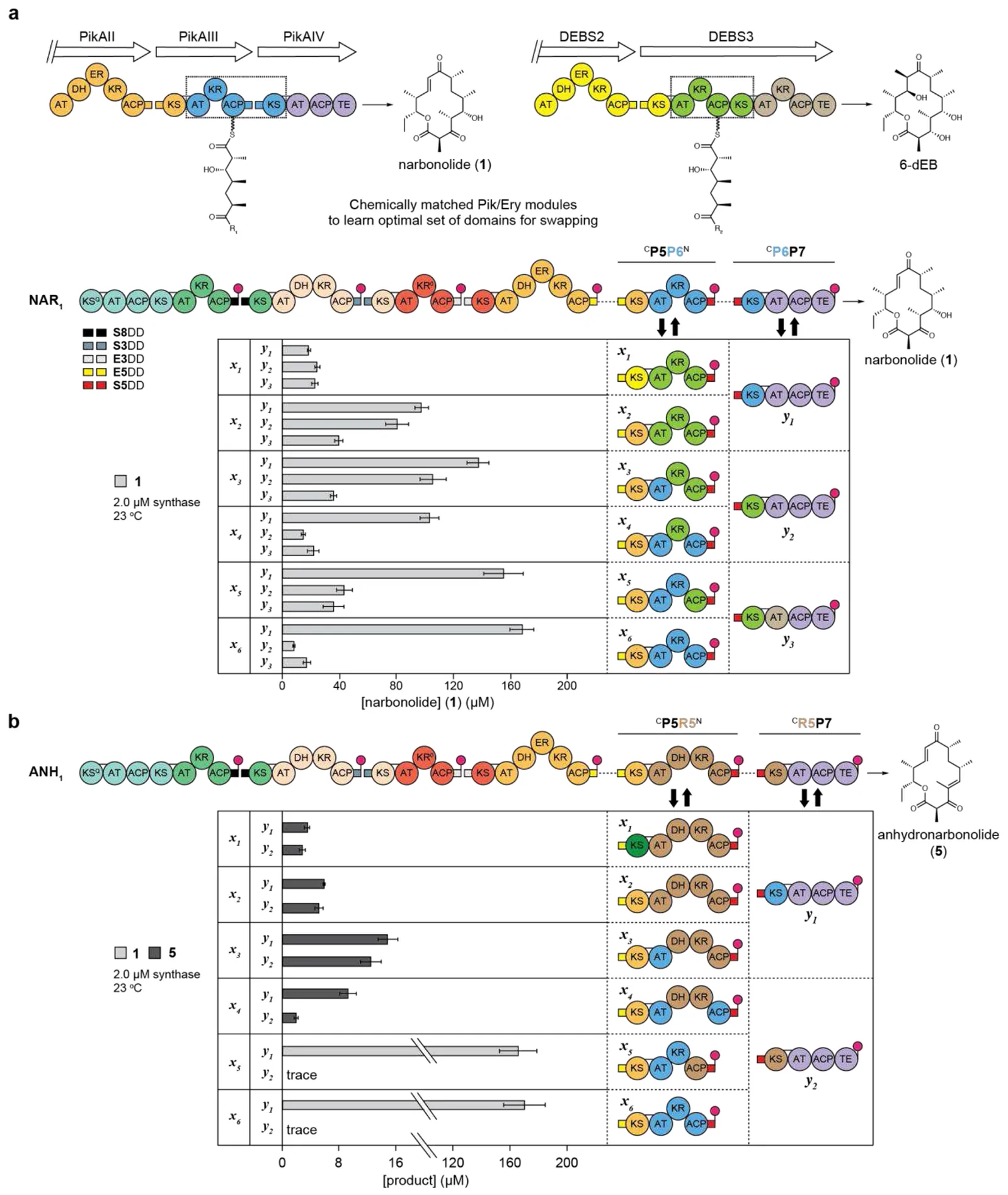
Optimizing domain compositions. a) Replacing **P6** of **P1**-**P2**-**P3**-**P4**-**P5**-**P6**-**P7** with **E6** entails swapping the AT, KR, ACP, and KS domains (**x_2_**, **y_2_**). The resulting synthase, **P1**-**P2**-**P3**-**P4**-**P5**-**E6**-**P7**, produces narbonolide at half the rate. However, when only KR, ACP, and KS are swapped (**x_3_**, **y_2_**), the resulting synthase produces narbonolide 25% faster than **P1**-**P2**-**P3**-**P4**-**P5**-**E6**-**P7**. b) Similarly, when only the KR, ACP, and KS domains of **P6** are replaced with the equivalent domains of **R5** (**x_2_**,**y_2_**), the resulting synthase is 2.5-fold more active than **P1**-**P2**-**P3**-**P4**-**P5**-**R5**-**P7** (**x_3_**,**y_2_**).

**Figure 5.**
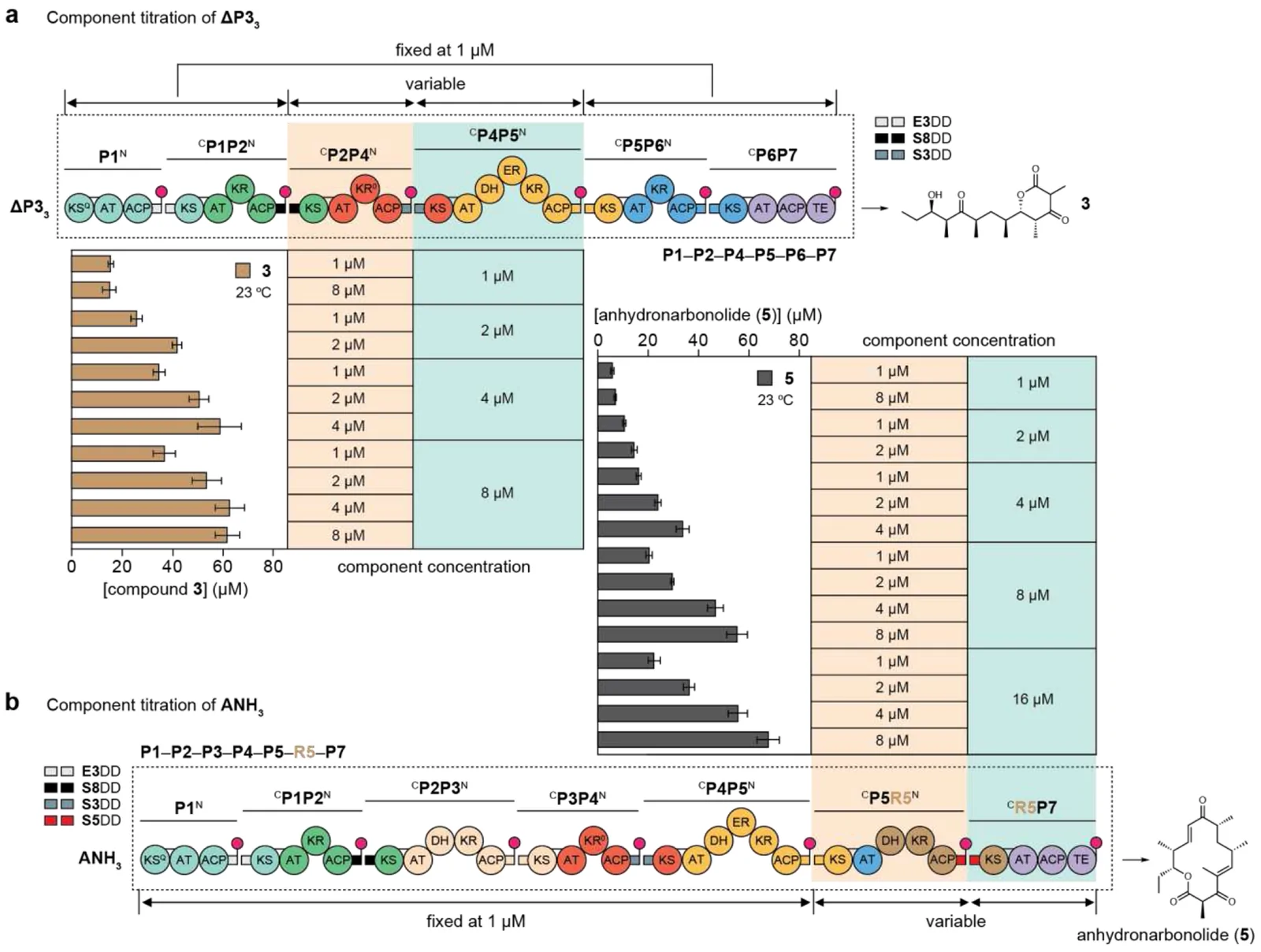
Optimizing polypeptide stoichiometry. a) Stoichiometry-tuning of the module-deleted synthase **ΔP3** shows that increasing the concentration of the polypeptide downstream of the deletion, ^**C**^**P4P5**^**N**^, increases production of hexaketide **3** more than increasing the concentration of the polypeptide from which the module was deleted, ^**C**^**P2P4**^**N**^. b) Stoichiometry-tuning of the module-swapped synthase **AHN** shows that increasing the concentration of both polypeptides containing the module swap, ^**C**^**P5R5**^**N**^ and ^**C**^**R5P7**, improves the production rate for heptaketide **5** (supplying 8 μM ^**C**^**P5R5**^**N**^ and 16 μM ^**C**^**R5P7** boosted productivity 12-fold).

**Figure 6.**
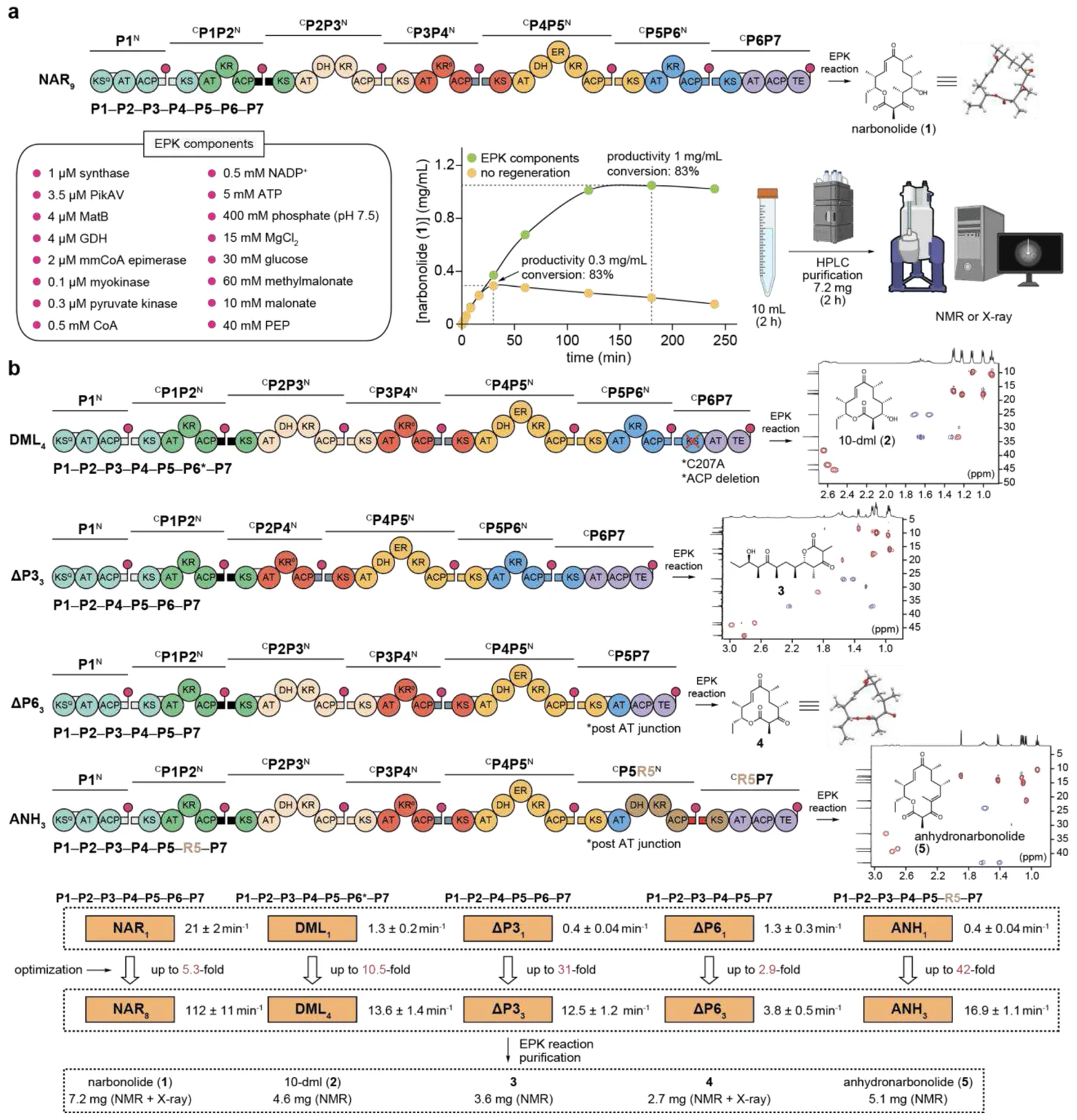
The Extender unit to PolyKetide (EPK) platform provides rapid, economic access to complex polyketides. a) The platform includes extender unit and NADPH regeneration systems that help convert malonate and methylmalonate to polyketide products. It was optimized by monitoring narbonolide (**1**) production from **NAR_9_**. The platform maintained high activity after 30 min, in contrast to reactions lacking regeneration systems. From a 2-hour, 10-mL reaction conducted at 23 °C, 7.2 mg pure narbonolide was obtained, enabling its crystal structure to be reported for the first time. b) EPK reactions were also performed with the 4 engineered variants of the pikromycin synthase that were optimized (up to 42-fold) for in vitro biocatalysis. They also yielded semi-preparative quantities of complex polyketides, which were structurally characterized by NMR spectroscopy and x-ray crystallography (Figs. S9–S35).

## Data Availability

All data are available through the Supplementary Information document and the Source Data file. The X-ray crystallographic coordinates for the structures reported in this study, **1** and **4**, have been deposited at the Cambridge Crystallographic Data Centre (CCDC), under deposition numbers 2513770 and 2578074, respectively. These data can be obtained free of charge from The Cambridge Crystallographic Data Centre via www.ccdc.cam.ac.uk/data_request/cif.
